# Identification and Evolution Analysis of the Genes Involved in the 20-Hydroxyecdysone Metabolism in the Mud Crab, *Scylla paramamosain*: A Preliminary Study

**DOI:** 10.3390/genes15121586

**Published:** 2024-12-10

**Authors:** Xin Jin, Lingbo Ma, Fengying Zhang, Linzi Zhang, Jinju Yin, Wei Wang, Ming Zhao

**Affiliations:** 1 Key Laboratory of East China Sea Fishery Resources Exploitation, Ministry of Agriculture, East China Sea Fisheries Research Institute, Chinese Academy of Fishery Sciences, 300 Jungong Road, Shanghai 200090, China; jx18036195921@163.com (X.J.); malb@ecsf.ac.cn (L.M.); zhangfy@ecsf.ac.cn (F.Z.); linzi9393@163.com (L.Z.); angela1322889698@163.com (J.Y.); wangw@ecsf.ac.cn (W.W.); 2 College of Fisheries and Life Science, Shanghai Ocean University, 999 Huchenghuan Road, Shanghai 201306, China

**Keywords:** *Scylla paramamosain*, Y-organ, *Neverland*, *CYP302a1*, *CYP307a1*, *CYP18a1*

## Abstract

Background: 20-Hydroxyecdysone (20E) is the most ubiquitous ecdysteroid (Ecd) and plays critical roles during the life cycle of arthropods. To elucidate the metabolism pathway of 20E in the economically important species, *Scylla paramamosain*, we conducted a comprehensive exploration of the genes involved in the 20E metabolism pathway. Methods: A comprehensive exploration of genes involved in the 20E metabolism pathway was conducted, including gene annotation, local blast using the *Drosophila* ortholog as query, and TreeFam ortholog genes identification. Bioinformatics and expression profiling of the identified genes were performed to assess their roles in the 20E metabolism of green mud crabs. Results: This experiment indicated that, except for *CYP306a1* and *CYP314a1*, all other ortholog genes involved in the *Drosophila* 20E metabolism can be found in the mud crab, suggesting that the function of these two genes might be replaced by other *CYP* genes or the “active” Ecd in mud crabs was not the 20E. All genes had the typical features of each gene family, clustered with the specific clade in the phylogenetic trees. In addition, all the identified genes had the highest expression level in the Y-organ, and sex-biased gene expression was observed in these genes. Conclusions: This study provided some valuable insights into the metabolism and diversity of ecdysteroids in crustaceans.

## 1. Introduction

Ecdysteroids are a diverse class of steroid hormones found in arthropods, such as insects and crustaceans. They play crucial roles in molting, development, and reproduction [1]. Over 500 ecdysteroid analogs have been identified, with 20-hydroxyecdysone (20E) being the most common and biologically active form across many species [2]. 20E is the primary molting hormone in most arthropods, serving as an active hormone that regulates various physiological processes, including gene expression, cell differentiation, and immune responses. These hormones are especially important in triggering the molting process during growth and development. Since the exoskeleton cannot expand, arthropods must shed their old exoskeleton to continue growing. During molting, the concentration of ecdysteroids increases periodically, reaching a threshold that initiates the molting process. Furthermore, ecdysteroids regulate other physiological processes such as reproduction and metabolism. Studies have shown a significant positive correlation between ecdysteroid levels and body weight during the pre-molt and inter-molt stages in the Chinese mitten crab (*Eriocheir sinensis*), further emphasizing the role of ecdysteroids in promoting the growth of this species. Additionally, research has found that female crabs have higher ecdysteroid levels than males, indicating that ecdysteroids play a role in female reproductive development [3]. In insect studies, ecdysteroids have been shown to be involved in ovarian development and vitellogenin production. Beyond their critical role in the development, metamorphosis, and reproduction of insects and crustaceans, ecdysteroids have other biological functions. For example, ecdysteroids cause role differentiation in bee social behavior by regulating gene expression; they induce programmed cell death in aging bee tissues, supporting the continued proliferation of imaginal disc cells, and they promote the synthesis of vitellogenin precursors and enhance ovarian development. Additionally, in the German cockroach (*Blattella germanica*), ecdysteroids regulate the synthesis pathway of aggregation pheromones during late life stages, though their potential regulatory role in bee pheromone synthesis remains to be explored further. Research also shows that injecting excess 20E into male moths inhibits and delays the release of spermatophores. Therefore, studying the genes involved in ecdysteroid metabolic pathways is essential to further elucidate these issues.

The synthesis pathway of 20-hydroxyecdysone (20E) in arthropods is a complex multi-step biosynthetic process that begins with cholesterol and involves the catalytic action of multiple enzymes. Initially, cholesterol is converted into intermediate molting hormone products through a series of hydroxylation and oxidation reactions. These hydroxylation reactions are primarily catalyzed by cytochrome P450 monooxygenases, including *CYP307a1* (Spook), *CYP306a1* (Phantom), *CYP302a1* (Disembodied), *CYP315a1* (Shadow), and *CYP314a1* (Shade), which introduce hydroxyl groups at different carbon positions. Subsequently, the hydroxylated intermediates undergo further oxidation to form α-keto compounds. Ultimately, through side-chain cleavage and cyclization, these intermediates are converted into the biologically active 20-hydroxyecdysone (20E). The Y-organ and the X-organ sinus gland complex are crucial neuroendocrine organs in crustaceans, with the Y-organ primarily secreting molting hormones. Crustaceans lack the ability to synthesize cholesterol on their own; hence, their molting hormones are converted from dietary cholesterol absorbed by the Y-organ [4]. Typically, the Y-organ sinus gland complex initially secretes the precursor of molting hormones, ecdysone, which is then transported into the hemolymph [5]. Under the action of 20-hydroxyecdysone dehydrogenase, ecdysone is transformed into the active molting hormone, 20-hydroxyecdysone (20E). The Y-organ was first identified as the main site for ecdysteroid synthesis by researchers Rudolph and Spaziani in the 1950s. Current research on the metabolic pathways of ecdysteroids is still in its infancy. The Neverland (Nvd) protein is essential for the de novo synthesis of ecdysteroids [6]. Nvd contains two conserved domains involved in enzyme catalysis: a Rieske [2Fe-2S] domain and a non-heme Fe(II)-binding motif [7]. The Rieske [2Fe-2S] domain acts as an electron acceptor and participates in electron transfer to other proteins, while the non-heme Fe(II)-binding motif, located at the C-terminus, is involved in oxygen binding [8]. In silkworms, it has been found that the *Nvd* gene is highly expressed in the prothoracic glands (PG), and a loss of the *Nvd* function in PG leads to larval growth arrest. The phenotype can be rescued by 20E or 7-dehydrocholesterol (7dC), indicating that *Nvd* plays a crucial role in the metabolism of cholesterol and steroid intermediates during ecdysteroid formation [7]. Additionally, knockdown experiments of *Nvd* in *Daphnia magna* embryos have shown that it can only complete one molting cycle normally, highlighting its importance in 20E metabolism [6]. The expression levels of *CYP302a1* and *CYP315a1* are closely related to the ecdysteroid titers in the hemolymph [2,9]. Moreover, these genes are highly expressed in molting steroidogenic tissues, with peak expression occurring just before the peak of ecdysteroid titers [10]. In crustaceans, only the *CYP302a1* gene has been cloned from *Portunus trituberculatus*, and its expression in the Y-organ is significantly higher than in other tissues. During the molting cycle, the expression pattern of *CYP302a1* in the Y-organ correlates with the changes in ecdysteroid titers, and its peak expression recedes the peak of molting hormone levels, indicating its essential role in the synthesis of molting hormones in this species [11]. In Drosophila, five essential hydroxylase genes for ecdysteroid biosynthesis (known as the Halloween genes) have been identified, including *CYP306a1*, *CYP302a1*, *CYP315a1*, *CYP307a1*, and *CYP314a1*. Molecular and biochemical studies have shown that the four hydroxylases, *CYP306a1*, *CYP302a1*, *CYP315a1*, and *CYP314a1* [12,13], play crucial roles in the final four steps of ecdysteroid synthesis, converting 5β-ketodiol to 20E. Additionally, mutations in genes involved in insect ecdysteroid biosynthesis can lead to abnormal embryonic development, which is caused by reduced ecdysteroid titers. Supplementing with 20E can restore normal development [7,14]. The identification of these genes provides a foundation for further research into the regulation of crustacean hormones.

*Scylla paramamosain*, commonly known as the mud crab, belongs to the family Portunidae and the genus *Scylla*. It is widely distributed along the southeastern coastal regions of China and is highly valued for its rich nutritional content and significant economic importance, making it one of the key marine economic crabs in China. With increasing interest from both consumers and researchers, the market demand for the mud crab continues to grow. Molting hormones play a crucial role in the growth and development of *S. paramamosain*, in addition to scientific feeding practices and optimal environmental conditions. The regulation of molting by these hormones is a key focus in crustacean research. An in-depth study of this issue could potentially provide a theoretical basis for enhancing the growth rate of mud crabs, significantly increasing their total yield during their growth cycle and reducing both abnormal growth rates and mortality due to molting failure. Currently, research reports on the metabolic pathways of molting hormones in *S. paramamosain* are scarce, making the analysis of its molting regulation pathways highly significant.

## 2. Materials and Methods

### 2.1. Ethics Statement

All animal experiments in this study were conducted in accordance with the relevant national and international guidelines. Our project was approved by the East China Sea Fisheries Research Institute. The mud crab (*S. paramamosain*) is not an endangered or protected species, and permission to perform experiments involving this species is not required in China.

### 2.2. Samples Collection

Healthy and high-quality female and male mud crabs were bred by the Genetic Breeding Innovation Team of *S. paramamosain* at the East China Sea Fisheries Research Institute of the Chinese Academy of Fishery Sciences, at the Hainan Qionghai base. Five female crabs in stage III of ovarian development and five male crabs in the spermatocyte stage were dissected. The hepatopancreas (Hep), Y-organ (YO), muscle (Mu), cerebral ganglion (CG), cuticle (Cu), thoracic ganglion (TG), hemocytes (He), ovary (OV), and testes (Te) were collected and placed into 2 mL DNAase/RNase-free sterile tubes (Kirgen Bioscience Co., Ltd., Shanghai, China) with *RNAhold^®^* (TransGen Biotech Co., Ltd., Beijing, China), stored at 4 °C overnight, and then transferred to a −80 °C ultra-low-temperature freezer. It is important to note that when collecting hemocytes, 1 mL of ACD anticoagulant (Sangon Biotech (Shanghai) Co., Ltd., Shanghai, China) should be drawn into a syringe, and approximately 1 mL of hemolymph should be collected from the unsclerotized membrane at the base of the leg, placed into a precooled 2 mL sterile tube, and immediately centrifuged at 4 °C, 8000 rpm for 10 min. The supernatant is discarded, and the precipitate is used for RNA extraction.

### 2.3. Genes Identification

Genes which might be involved in 20E metabolism were firstly identified using the gene annotation information from the protein coding gene database, which was predicted and built by both transcriptome and genomic data. For those genes which could not be identified, we used the local blast method that uses the orthologs from the *Drosophila melanogaster* as the query sequences, which were downloaded from the KEGG database. In addition, TreeFam [15] ortholog genes identification was also used to examine the presence or not in other animals’ genomes, including *Daphnia pulex* [16], *E. sinensis* [17], *P. trituberculatus* [18], *S. paramamosain* [15], and *Litopenaeus vannamei* [19]. Protein coding gene datasets of these species were download from the NCBI genome database (https://www.ncbi.nlm.nih.gov/datasets/genome/, accessed on 28 March 2024) or the GWH database (https://ngdc.cncb.ac.cn/gwh/, accessed on 28 March 2024). All identified genes were blasted again in the Non-Redundant Protein Sequence Database and InterPro database to verify their function annotation results. The CDS sequences of these genes identified in the mud crabs were verified using the PCR and sanger sequencing, and the primers are provided in Table 1.

### 2.4. The Extraction of Total RNA

The extraction of total RNA from the samples was conducted entirely in a fume hood (excluding the centrifugation steps). Disposable sterile masks and gloves were worn throughout the process, with gloves changed regularly to avoid contamination by exogenous RNase. Since chloroform and RNA lysis buffer are toxic reagents, caution should be taken to prevent contact with skin and clothing to avoid unnecessary harm. Additionally, it is important to perform the extraction process at maximum speed whenever possible.

Total RNA extraction was performed using the EZ-10 DNAaway RNA Mini-Preps Kit (Sangon Biotech (Shanghai) Co., Ltd., Shanghai, China), following the instructions provided in the kit’s manual. After RNA extraction, 3 μL of RNA was used for agarose gel electrophoresis and spectrophotometric analysis to evaluate its quality and concentration, while the remaining samples were stored at −80 °C.

### 2.5. Primer Design

The primers used in this study were designed using Primer5.0 software (https://www.premierbiosoft.com/primerdesign/, accessed on 2 April 2024) based on the obtained sequences and synthesized by Shanghai Jieli Bio-Tech Co., Ltd., Shanghai, China. The primers used in the experiments are listed in the table (Table 1).

### 2.6. Multiple Sequence Alignment Analysis

The open reading frames of the gene sequences were identified and translated using ORF Finder (http://www.ncbi.nlm.nih.gov/gorf/gorf.html, accessed on 3 April 2024). BLAST (https://blast.ncbi.nlm.nih.gov/Blast.cgi, accessed on 3 April 2024) analysis was then performed in the Nr database for preliminary gene annotation. Amino acid sequences of the *Neverland*, *CYP302a1*, *CYP315a1*, *CYP307a1*, and *CYP18a1* genes from multiple species were downloaded from GenBank (https://www.ncbi.nlm.nih.gov/genbank, accessed on 3 April 2024) and aligned using DNAMAN10.0 software (https://www.lynnon.com/dnaman.html, accessed on 3 April 2024) to analyze their similarities with other species. The ExPASy ProtParam tool (https://web.expasy.org/protparam/, accessed on 3 April 2024) was used to predict the molecular weight and isoelectric point of the proteins. The presence of signal peptides in the proteins was predicted using the SignalP 5.0 Server (http://www.cbs.dtu.dk/services/SignalP/, accessed on 5 April 2024), and the presence of transmembrane regions was predicted using the online TMHMM tool (https://services.healthtech.dtu.dk/services/TMHMM-2.0/, accessed on 5 April 2024). The SWISS-MODEL online tool (https://swissmodel.expasy.org/, accessed on 5 April 2024) is used to predict the tertiary structural modeling of ecdysone-related gene proteins. To analyze the conserved motifs related to genes involved in ecdysteroid metabolism, the MEME tool (http://meme-suite.org, accessed on 6 April 2024) was used for sequence analysis. The gene sequences utilized were obtained from relevant databases or experimental data.

### 2.7. Evolution Analysis

Amino acid sequences of Neverland, CYP307a1, CYP315a1, CYP18a1, and other genes involved in the synthesis and metabolism of 20E from the P450 family, including CYP302a1, CYP307a1, and CYP314a1, which were downloaded from the GenBank database for various arthropod species. The phylogenetic tree was constructed using MEGA11 (https://www.megasoftware.net/, accessed on 15 April 2024) and the evolutionary tree was annotated and refined using the online tool Interactive Tree of Life (https://itol.embl.de, accessed on 15 April 2024).

### 2.8. Tissue Expression Analysis

Using 0.2 μg total RNA as a template, the first-strand cDNA was synthesized with the EasyScript^®^ One-Step RT-PCR SuperMix (TransGen Biotech), following the manufacturer’s instructions. The qRT-PCR experiment uses 18S as the reference gene, with three replicates per group. The expression levels of the genes *Neverland*, *CYP302a1*, *CYP315a1*, *CYP307a1*, and *CYP18a1* are measured in nine tissues: hepatopancreas (Hep), Y-organ (YO), muscle (Mu), gill (Gi), cuticle (Cu), thoracic ganglion (TG), hemocytes (He), ovary (OV), and testes (Te). The reaction system is 10 μL in total: 5 μL of Tip Green qPCR SuperMix (TransGen Biotech), 1 μL of cDNA template (80 ng/μL), 0.5 μL each of the forward (10 μM) and reverse primers (10 μM), and 3.0 μL of ddH₂O. The reaction conditions are as follows: pre-denaturation at 95 °C for 2 min, denaturation at 95 °C for 15 s, annealing at 60 °C for 30 s, extension at 70 °C for 30 s, 35 cycles, followed by the melt curve provided by the qRT-PCR kit. The results are calculated using the 2^−ΔΔCT^ (Livak) method [20].

### 2.9. Statistical Analysis

The data were analyzed using the one-way ANOVA method, and the post hoc test was carried out using a Tukey multiple comparison test using SPSS 22.0 (https://www.ibm.com/cn-zh/spss, accessed on 16 March 2024). The differences were considered significant at *p* < 0.05.

## 3. Results

### 3.1. Presence and Basic Information of Genes Related to Molting Hormones in Crustaceans

With the development of high-throughput sequencing technology, more and more cytochrome P450 genes have been identified, and the possession of ecdysone metabolism-related genes in different species has been described (see Table 2 for detailed in-formation).

In this paper, we focus on four genes related to the 20E metabolic pathway in the mud crab (*S. paramaosain*): *Neverland*, *CYP302a1*, *CYP307a1*, *CYP315a1* and *CYP18a1* (Table 3).

### 3.2. Secondary and Tertiary Structure Prediction of 20E Metabolism Proteins

#### 3.2.1. Secondary Structure Prediction of 20E Metabolism Proteins

The Neverland protein has an α-helix content of 34.56%, with the highest random coil content at 44.06%, indicating a relatively flexible structure that may facilitate dynamic protein–protein interactions. In contrast, CYP307a1, CYP315a1, and CYP302a1 exhibit higher α-helix contents of 42.66%, 48.78%, and 45.85%, respectively, suggesting greater structural stability and regularity. Notably, CYP315a1 has the highest α-helix content at 48.78%, with relatively low proportions of β-turn and random coil, indicating a more rigid structure that may be associated with its stable enzymatic activity in the ecdysone pathway. Similarly, CYP18a1 shows an α-helix content of 47.81%, comparable to CYP315a1, indicating a high degree of regularity and stability in its secondary structure (Table 4).

#### 3.2.2. Tertiary Structural Modelling of 20E Metabolism Proteins

**Figure 1:**
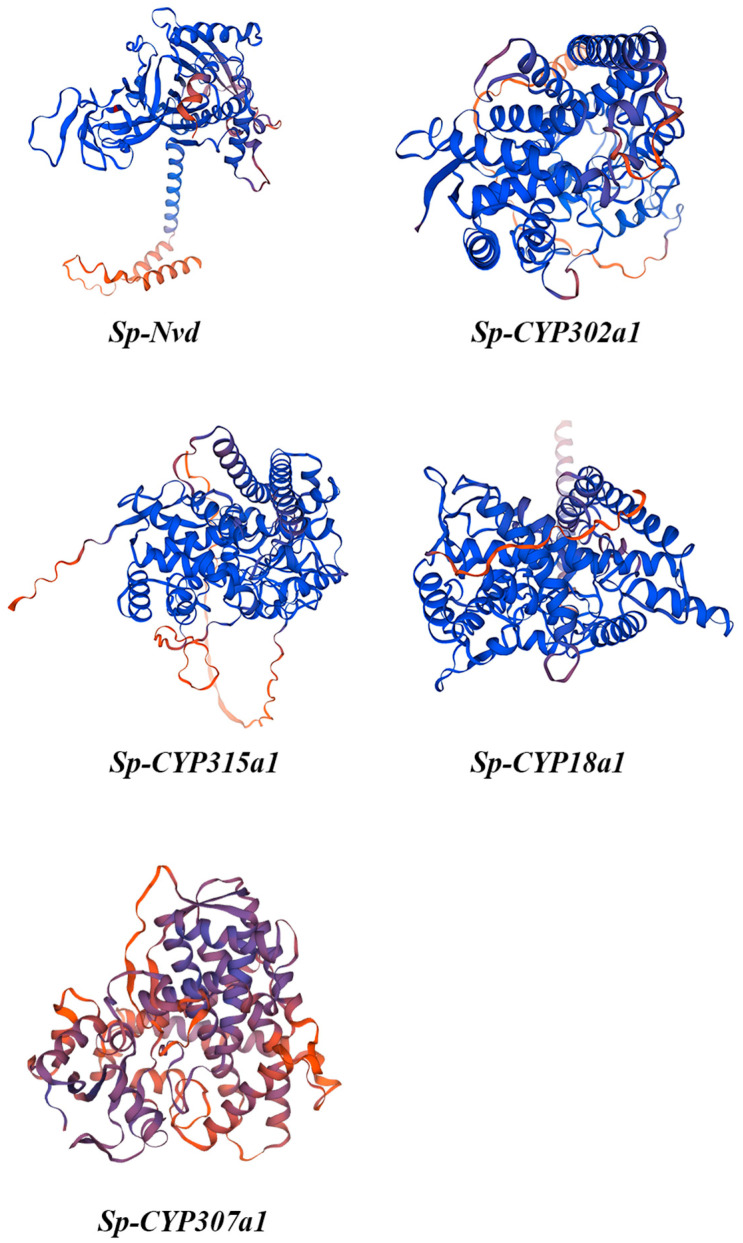
Tertiary structural modelling of 20E metabolism proteins.

In the tertiary structures of the proteins, the blue sections represent α-helices, while the red sections represent random coils, illustrating the overall folding pattern of each protein. These structures align perfectly with the secondary structure composition table. The proportions of α-helices and random coils for each protein are mutually confirmed between the image and the table (Figure 1).

### 3.3. Evolutionary Analysis of Gene Families

#### 3.3.1. Multiple Sequence Alignment

**Figure 2:**
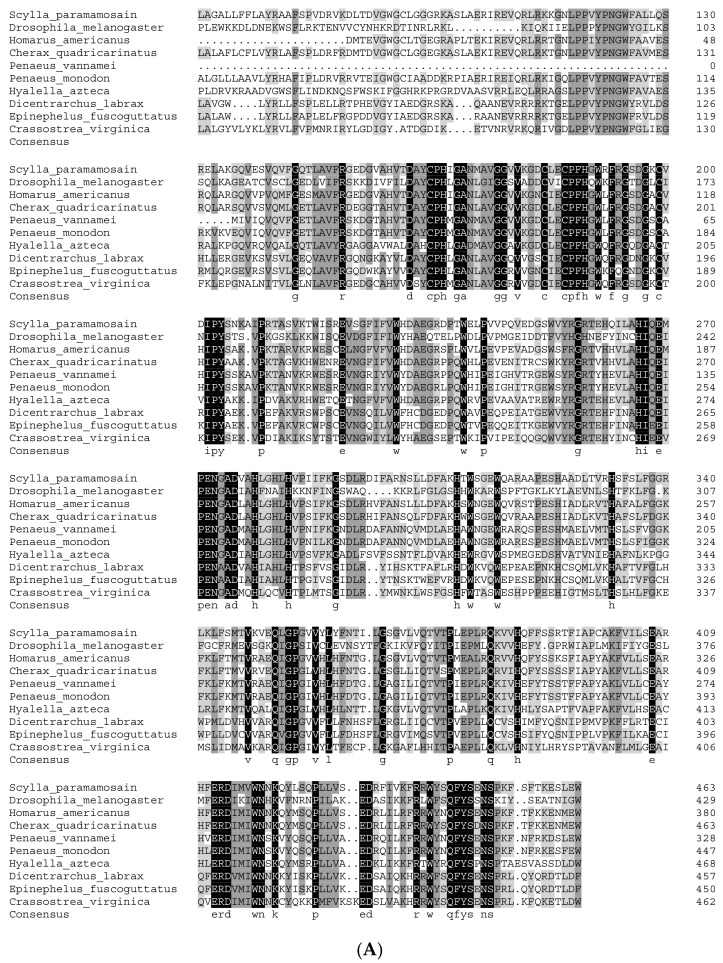

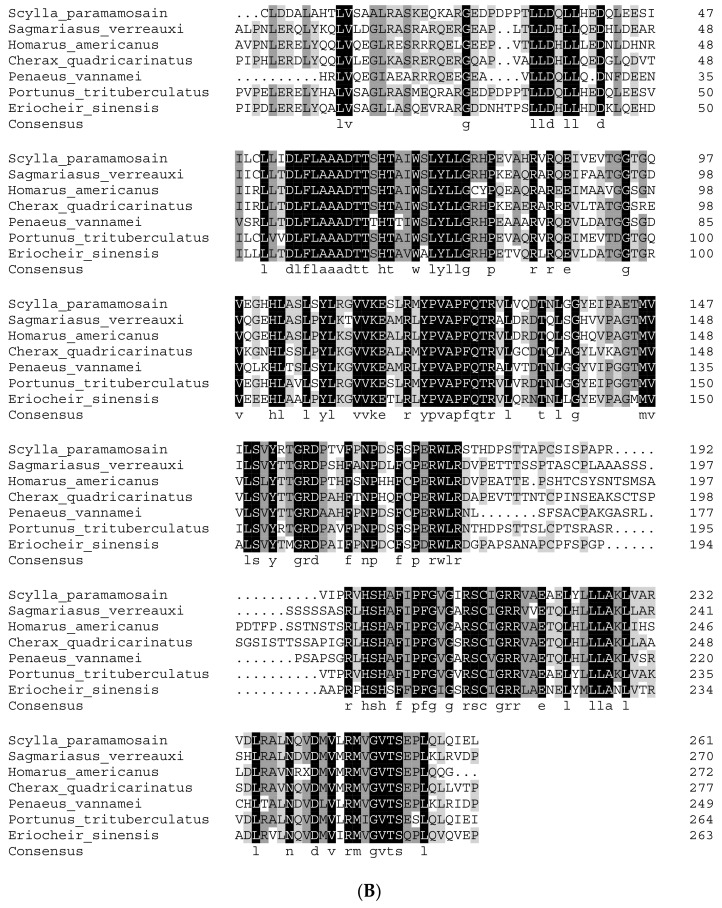

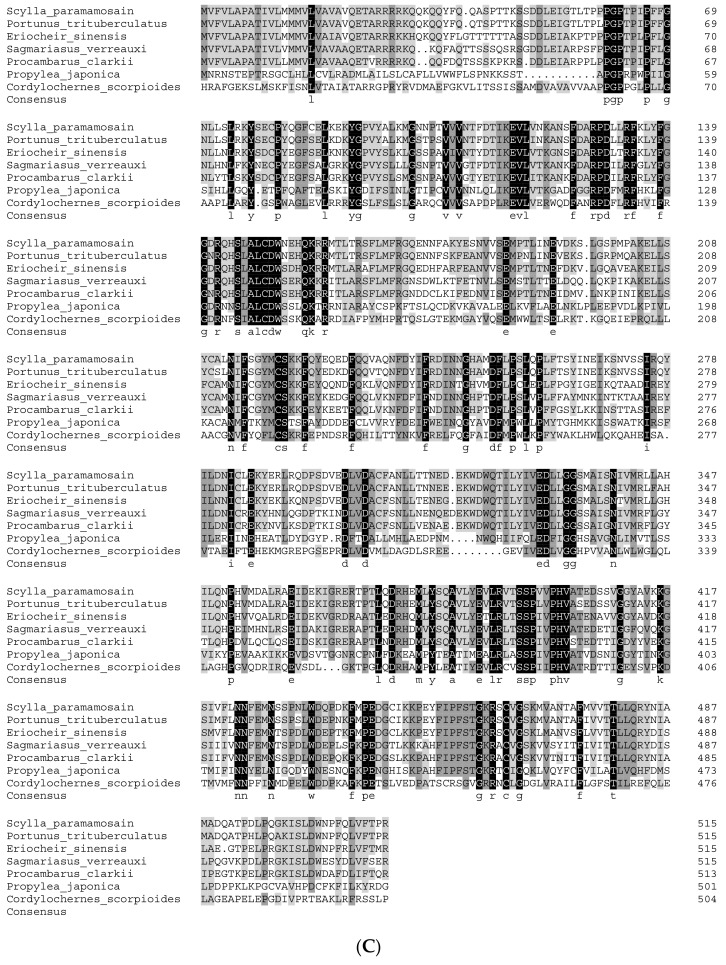

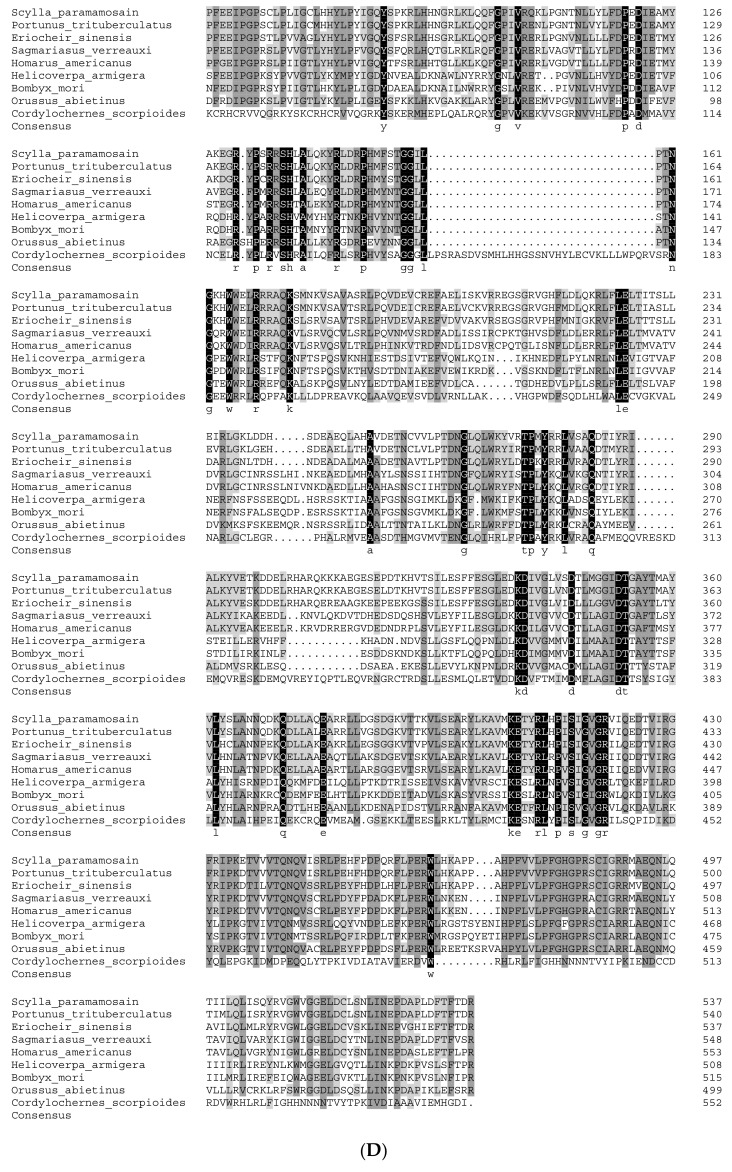

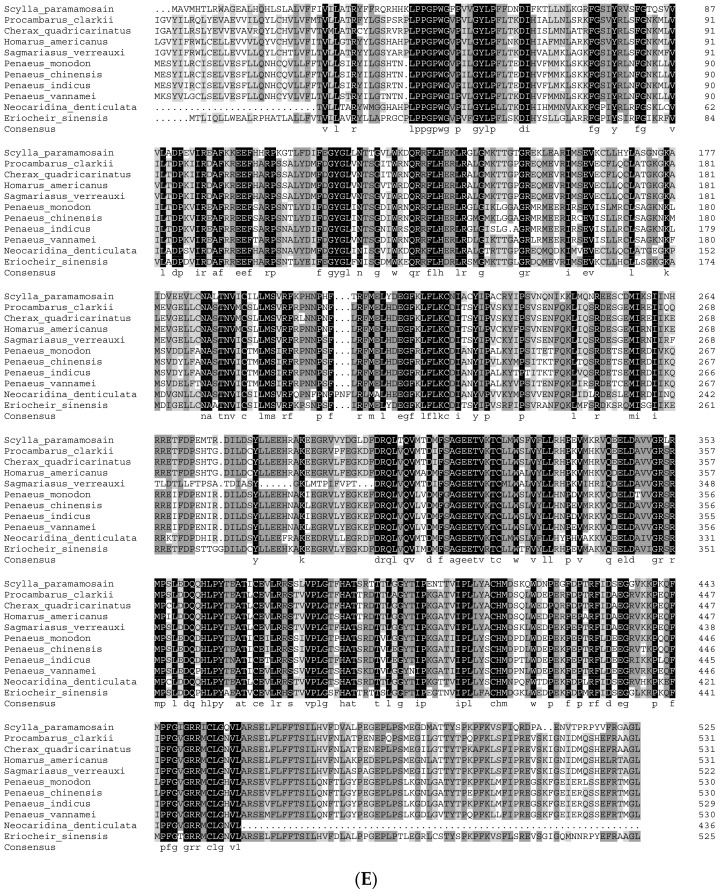
Multiple sequence alignment analysis. Sequences with 100% similarity are highlighted with a black background, sequences with over 75% similarity are highlighted with a dark gray background, and sequences with over 50% similarity are highlighted with a light gray background. (**A**) Multiplex protein sequences alignment of Nvds. (**B**) Multiplex protein sequences alignment of CYP315a1. (**C**) Multiplex protein sequences alignment of CYP307a1. (**D**) Multiplex protein sequences alignment of CYP302a1. (**E**) Multiplex protein sequences alignment of CYP18a1.

The amino acid sequences used in the multiple sequence alignment are listed in Table S1. Multiple sequence alignment analyses revealed that the amino acid sequence of Sp-Nvd has the highest homology with *Penaeus vannamei*, at 70%, followed by *Penaeus monodon* and *Hyalella azteca*, with homologies of 67% and 58%, respectively (Figure 2A). The amino acid sequences of Sp-CYP315a1 show the highest similarity with *Sagmarisus verreauxi* at 91%, followed by *Homarus americanus* and *Cherax quadricarinatus*, with homologies of 56% and 46%, respectively (Figure 2B). The amino acid sequence of Sp-CYP307a1 shows the highest homology with *P. trituberculatus*, followed by *E. sinensis*, with homologies of 84% and 57%, respectively (Figure 2C). The amino acid sequence of Sp-CYP302a1 has the highest homology with *P. trituberculatus* at 65%, followed by *E. sinensis* and *P. vannamei*, with homologies of 64% and 63%, respectively (Figure 2D). The amino acid sequence of Sp-CYP18a1 shows the highest homology with *Procambarus clarkii*, followed by *C. quadricarinatus*, with homologies of 52.04% and 45.16%, respectively (Figure 2E).

#### 3.3.2. Evolutionary Tree

**Figure 3:**
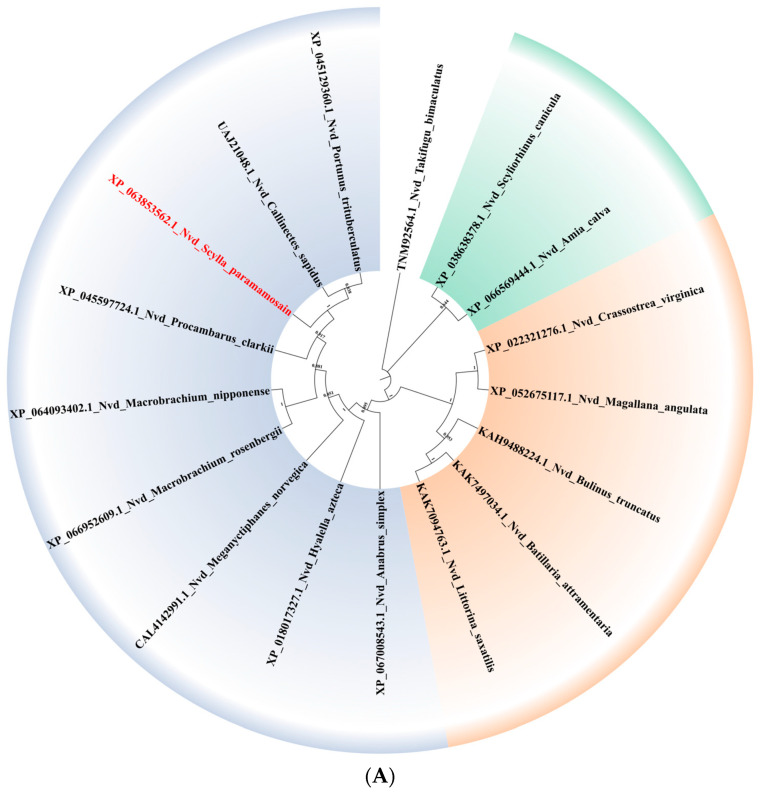

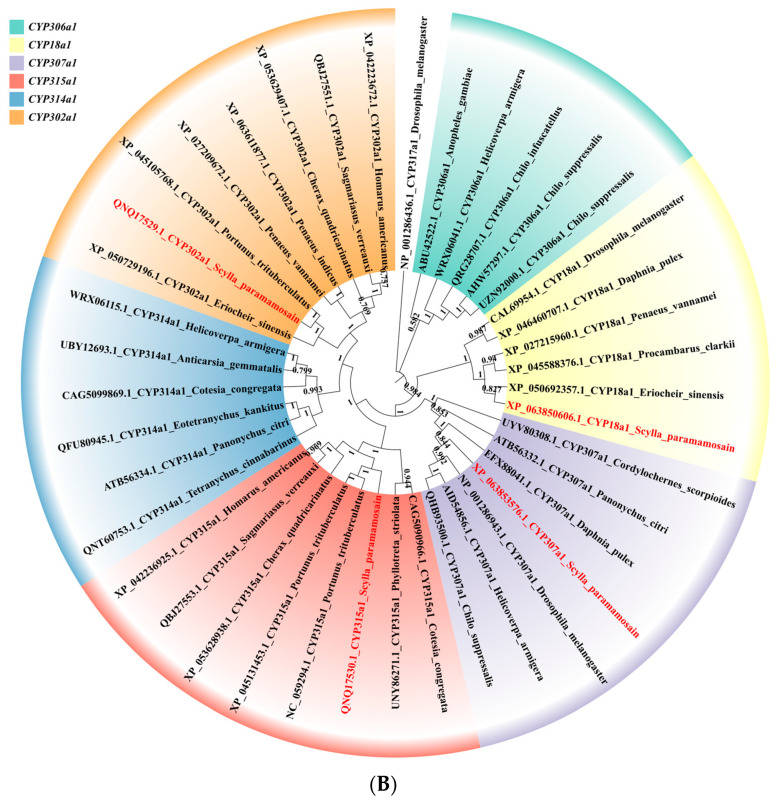
Evolutionary tree. (**A**) The phylogenetic tree of Neverland in different species. The tree is constructed using the Neighbor-Joining (NJ) method. The size of the circle on the branch represents the bootstrap support rate. (**B**) The phylogenetic tree of P450 family genes involved in 20E metabolism. The trees is constructed using the Neighbor-Joining (NJ) method. The size of the circle on the branch represents the bootstrap support rate.

Two phylogenetic trees were constructed, each including seven major gene categories: Neverland, CYP306a1, CYP307a1, CYP315a1, CYP314a1, CYP302a1, and CYP18a1. The Sp-Nvd gene clusters with other Nvd genes and shows the closest evolutionary relationship with the Chinese mitten crab (*E. sinensis*), followed by the American gammarid (Figure 3A). The amino acid sequences of each distinct CYP gene are grouped together. The Sp-CYP18a1 gene clusters with the CYP18a1 genes of crustaceans such as *D. pulex*, *P. vannamei*, and *P. clarkii*, exhibiting a high degree of sequence homology. The Sp-CYP307a1 gene clusters with CYP307a1 genes from other arthropods such as *D. pulex* and *Panonychus citri*, but is relatively distant from the branch of *D. melanogaster*. The Sp-CYP315a1 gene forms a highly supported branch with CYP315a1 genes from various arthropod species, including *D. melanogaster*. The Sp-CYP302a1 gene shows a high level of evolutionary similarity with the CYP302a1 genes of insects such as *D. melanogaster* and *Helicoverpa armigera* (Figure 3B).

### 3.4. The Results of the Relative Expression of Genes in Different Tissues

**Figure 4:**
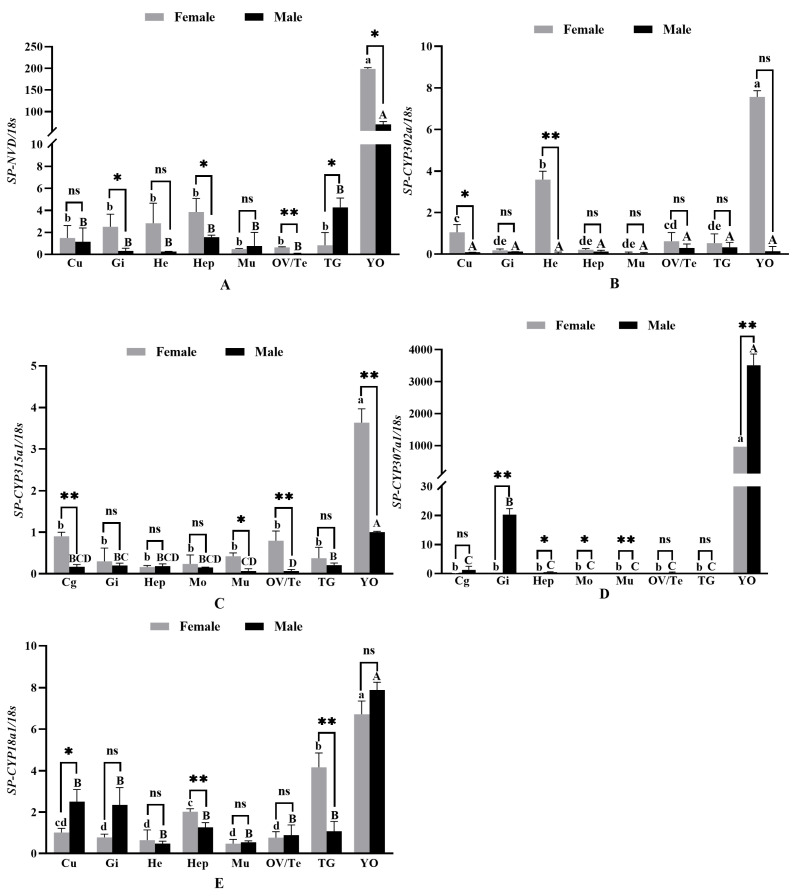
The results of the relative expression of genes in different tissues. Note: Hep: hepatopancreas; YO: Y-organ; Mu: muscle; Gill: gill; Cu: cuticle; TG: thoracic ganglion; He: hemocytes; OV: ovary; Te: testes. “*” indicates significant difference between the same or related tissues in different gender (*p* < 0.05); “**” indicates extremely significant difference between the same or related tissues in different gender (*p* < 0.01); “ns” indicate no significant difference. (**A**–**E**) Different lowercase or uppercase letters indicate significant differences among the different tissues in the same gender.

Quantitative results of *Sp-Nvd* showed that in the Y organ (YO), Gill (Gi), hepatopancreas (Hep), and antennal gland (TG), the expression in females was significantly higher than in males (*p* < 0.05), while no significant gender differences were observed in other tissues, including the epidermis (Cu), hemocytes (He), muscle (Mu), and gonads (OV/Te). Particularly in the Y organ, the gene expression level in females was much higher than that in males (Figure 4A). The *Sp-CYP302a1* gene showed considerable variation in expression across different tissues, with a relatively similar tissue distribution in both female and male crabs. It was expressed in all detected tissues, but the expression level in the Y organ (YO) was significantly higher than in other tissues (*p* < 0.05), while expression levels in other tissues were relatively low. In the hemocytes (He) and epidermis (Cu), female expression was significantly higher than male expression (*p* < 0.05). No significant differences between females and males were observed in other tissues, including the Gill (Gi), hepatopancreas (Hep), muscle (Mu), gonads (OV/Te), antennal gland (TG), and Y organ (YO) (Figure 4B). In the epidermis (Cu), muscle (Mu), and gonads (OV/Te), the expression of the *Sp-CYP315a1* gene was significantly higher in females than in males (*p* < 0.05 and *p* < 0.01). Especially in the Y organ (YO), female expression was significantly higher than male expression (*p* < 0.01). In the Gill (Gi) and Y organ, male expression levels were significantly lower than those in females (Figure 4C). For the *Sp-CYP307a1* gene, female expression levels were significantly lower than male levels in all detected tissues. In the Gill (Gi) and Y organ (YO), male expression was significantly higher than female expression (*p* < 0.01), exhibiting particularly high levels in the Y organ. Additionally, in the hepatopancreas (Hep), muscle (Mu), and gonads (OV/Te), male expression was higher than female expression (*p* < 0.05) (Figure 4D). For the *Sp-CYP18a1* gene, expression in the epidermis (Cu) and hepatopancreas (Hep) was significantly lower in females than in males (*p* < 0.05 and *p* < 0.01), whereas in the antennal gland (TG), the expression level in females was significantly higher than in males (*p* < 0.01). No significant differences were observed in other tissues such as the Gill (Gi), hemocytes (He), muscle (Mu), gonads (OV/Te), and Y organ (YO). In the epidermis and hepatopancreas, male expression was significantly higher than female expression (Figure 4E).

## 4. Discussion

In *S. paramamosain*, the absence of *CYP306a1* and *CYP314a1*, both of which are typically involved in the synthesis and metabolism of ecdysteroids, suggests an interesting evolutionary divergence from other arthropods where these genes are closely associated with the molting process. This absence indicates that *S. paramamosain* may have evolved alternative mechanisms to regulate its hormone metabolism process, possibly relying on other hormonal pathways or enzymes. For example, instead of utilizing these cytochrome P450 genes, *S. paramamosain* might regulate molting through other key signaling pathways such as the MAPK pathway, which has been shown to play a significant role in developmental regulation. This functional redundancy and pathway diversity might have rendered *CYP306a1* and *CYP314a1* unnecessary for the species. These findings highlight the adaptability and complexity of metabolic pathways in crustaceans, where multiple mechanisms can evolve to support essential physiological processes like molting [21,22]. *S. paramamosain* inhabits coastal regions with significant fluctuations in salinity, and its long-term adaptation to these environmental changes has led to unique evolutionary developments in its metabolic system. Studies have shown that when responding to sudden environmental stressors, *S. paramamosain* shifts its metabolic focus from steroid metabolism to amino acid and energy metabolism. This metabolic shift likely diminishes the role of *CYP306a1* and *CYP314a1*, as the rebalancing of the metabolic system makes them less essential for survival under such conditions. This suggests that *S. paramamosain* has evolved alternative pathways and mechanisms to prioritize energy and amino acid metabolism over steroid metabolism in fluctuating environments, reflecting its adaptability to saline changes. The downregulation or loss of *CYP* genes like *CYP306a1* and *CYP314a1* may be part of a broader evolutionary strategy to enhance survival in extreme or rapidly changing conditions [23,24].

The sequence alignment results show that the homology of the mud crab (*S. paramamosain*) CYP18a1 and CYP307a1 genes with those of *D. melanogaster* is relatively low, at 39.60% and 49.44%, respectively. Typically, such a low homology indicates that these genes may have undergone functional diversification during evolution. A phylogenetic analysis reveals that these two genes are located on different branches, distant from the CYP gene clade of *D. melanogaster* and other insects, suggesting a significant divergence in their functional roles across species. CYP307a1 is closely associated with the regulation of ecdysteroids in some arthropods. Although its primary role may be in ecdysteroid biosynthesis, studies have also shown that it plays a critical role in the metabolic balance of these hormones. Specifically, in crustaceans, the regulatory pathway involving the CYP307a1 gene suggests its involvement in feedback mechanisms that regulate the degradation or metabolism of ecdysteroids during the molting process. This indicates a possible shift from its role in synthesis towards functions related to hormonal homeostasis [25]. CYP18a1 is closely linked to the degradation of 20-hydroxyecdysone (20E) in insects and other arthropods. This gene plays a crucial role in regulating ecdysteroid hormone levels, particularly by degrading 20E to control its concentration during specific developmental stages. In many insects, CYP18a1 is considered one of the primary enzymes involved in the metabolism of ecdysteroids, directly impacting the molting process. By modulating the concentration of 20E, CYP18a1 ensures that hormonal levels are appropriate for the insect’s developmental stage, thereby influencing growth and other critical physiological processes [26].

The *Nvd* (*Neverland*) gene is an important gene in the steroid synthesis pathway, involved in the synthesis of ecdysteroids in crustaceans [27]. By catalyzing the conversion of phytosterols (such as sitosterol and cholesterol) into 7-dehydrocholesterol, the *Nvd* gene initiates the ecdysteroid synthesis pathway. The high expression of the *Nvd* gene in the Y organ (YO) suggests that it may play a greater role in hormonal regulation during the female reproductive cycle. In shrimp and crab species, the RNA interference (RNAi)-mediated knockdown of *Nvd* gene expression results in a significant inhibition of ovarian development in females, suggesting that *Nvd* plays a critical role in the regulation of female reproductive processes [28]. As the main endocrine organ in crustaceans, the Y organ is responsible for the synthesis and secretion of ecdysteroids, thereby regulating molting and developmental processes. Studies have found that the expression level of the *Nvd* gene in the YO of females is much higher than that in males, indicating that this gene may play a more significant role in hormonal regulation in females. The reproductive cycle of females may require stronger endocrine regulation, especially during ovarian development and spawning. This difference may be related to their need for more ecdysteroids to support reproduction and developmental processes [29]. This phenomenon has also been found in other crustacean species. For example, in shrimps, researchers have observed that the activity of the Y organ in females during the breeding season is significantly higher than in males [30]. In female individuals, the high expression of the *Nvd* gene is likely associated with the active state of the endocrine system during the reproductive period. The reproductive cycle of crustaceans is regulated by steroid hormones, the synthesis of which depends on the continuous expression of *Nvd*. This study revealed that the expression levels of *Nvd* in the Gill (Gi), hepatopancreas (Hep), and thoracic ganglion (TG) are significantly higher in females compared to males. As the primary organs for digestion and nutrient absorption, the elevated expression of *Nvd* in the stomach and hepatopancreas may support an enhanced energy metabolism, thereby providing sufficient nutritional reserves for reproductive activities. Additionally, the hepatopancreas, being a major energy storage organ in crustaceans, likely plays a crucial role in meeting the energy demands during the reproductive period through the elevated expression of *Nvd* [31]. Despite the lack of significant sex-specific differences in *Nvd* gene expression in tissues such as the epidermis, heart, muscle, and gonads, this does not preclude its important biological functions in these tissues. On the contrary, it may reflect the homeostasis of gene expression in these tissues in both male and female individuals, suggesting that it is not significantly influenced by reproductive or sex-specific regulatory factors. Specifically, the expression of *Sp-Nvd* in the gonads (OV/Te) showed no significant sex differences, which may imply that this gene does not directly participate in sex differentiation or germ cell development within the gonads. The *CYP18a1* enzyme exerts a negative regulatory effect on steroid hormone signaling by hydroxylating 20-hydroxyecdysone (20E) into 20-hydroxyecdysonoic acid, thereby inactivating 20E [32]. The expression of the *CYP18a1* gene in various tissues of the mud crab (*S. paramamosain*) exhibits significant sex-specific differences. For instance, *CYP18a1* expression in the cuticle (Cu) and hepatopancreas (Hep) is significantly higher in males compared to females, which may be associated with the more frequent molting cycles and higher energy metabolism demands in males. Male individuals need to complete the molting process more rapidly to facilitate growth, particularly to gain an advantage in competition. Therefore, the elevated expression of *CYP18a1* contributes to the accelerated degradation of ecdysteroids, thereby expediting the molting process and providing sufficient energy for growth. This observation aligns with the characteristic of male crustaceans generally requiring a faster growth rate to achieve competitive superiority [33]. The Y organ (YO) is the primary site for ecdysteroid synthesis, with several signaling pathways, such as Wnt, Hedgehog, and Notch, playing crucial regulatory roles in its function [34]. However, no significant sex differences were observed in the Y organ, suggesting the presence of a tightly regulated balancing mechanism in the control of hormone synthesis and metabolism, ensuring that both male and female individuals meet the requirements for normal development and reproduction. Additionally, the inhibitory hormones MIH (molt-inhibiting hormone) and CHH (crustacean hyperglycemic hormone) may exert negative feedback regulations on the Y organ, potentially affecting the expression and activity of *CYP18a1*, and thereby influencing the metabolism of ecdysteroids [35]. The enzyme encoded by *CYP315a1* is a key cytochrome P450 monooxygenase involved in the ecdysteroid biosynthesis pathway, responsible for hydroxylating ecdysone into 20-hydroxyecdysone (20E), which is the most biologically active ecdysteroid in crustaceans and insects [26]. In insects, *CYP315a1* (also known as “Shade”) has been extensively studied. Riddiford et al. identified the function of *CYP315a1* in *Drosophila* through molecular and biochemical approaches, confirming its crucial role in the final step of ecdysteroid synthesis [36]. This study found that female *S. paramamosain* exhibited higher *Sp-CYP315a1* expression levels in the Y organ, cuticle, muscle, and gonads, which may be closely related to their reproductive physiological demands. The Y organ is the primary site of ecdysteroid synthesis, and the elevated expression of *CYP315a1* likely enhances the synthesis of 20-hydroxyecdysone (20E), providing essential hormonal support for ovarian development and oocyte maturation [37]. Ecdysteroids are believed to play a crucial role in vitellogenesis in crustaceans, promoting the synthesis of vitellogenin and the growth of oocytes [37]. The expression pattern of the *Sp-CYP307a1* gene showed that females had significantly lower expression levels compared to males across all tested tissues. This sex difference aligns with the general phenomenon of the sex-biased expression of P450 genes in arthropods. The P450 gene family in arthropods has a wide range of metabolic functions, including steroid hormone synthesis and detoxification [38]. In males, the high expression of *Sp-CYP307a1* in the Gill (Gi), Y organ (YO), hepatopancreas (Hep), muscle (Mu), and gonads (OV/Te) suggests that this gene may play a critical role in androgen synthesis and metabolism. The significantly higher expression in the Y organ, a key site for ecdysteroid synthesis, further underscores the function of *CYP307a1* in hormonal regulation in males. The Y organ is the primary site of ecdysteroid synthesis in arthropods, and the elevated expression of *Sp-CYP307a1* in this tissue implies its central role in the ecdysteroid biosynthetic pathway [39]. Previous studies on various arthropods, such as Anopheles gambiae and Bombyx mori, have further supported the importance of *CYP307a1* in steroid hormone biosynthesis and the regulation of ecdysteroids [40]. In these species, the expression levels of *CYP307a1* are associated with male development, particularly in the gonads and other reproductive-related tissues, which is consistent with the findings of high expression in males in this study. These results suggest that *CYP307a1* is crucial for maintaining male-specific reproductive and growth functions. Additionally, studies on the white-backed planthopper (*Sogatella furcifera*) have shown that the inhibition of *CYP307a1* leads to severe developmental defects, further demonstrating its critical role in molting and developmental processes [41]. By inhibiting the expression of this gene through RNA interference, researchers observed severe molting disorders and developmental arrest, further confirming the indispensable role of *Sp-CYP307a1* in the molting process of arthropods. The expression levels of the *Sp-CYP302a1* gene in different tissues of *S. paramamosain* showed significant variation, with similar tissue distribution patterns between male and female individuals. However, there were significant differences in the expression between males and females in certain specific tissues, suggesting that this gene may play an important role in sex-related physiological functions. Notably, the expression level of *Sp-CYP302a1* in the Y organ (YO) was significantly higher than in other tissues, which is consistent with its central role in ecdysteroid synthesis [42]. The Y organ is the primary site for ecdysteroid synthesis; therefore, the high expression of the *Sp-CYP302a1* gene in this tissue likely plays a crucial role in promoting ecdysteroid biosynthesis. Furthermore, this elevated expression may reflect the similarities in the molting regulation between males and females, as ecdysteroids are essential for maintaining normal development and the molting process in both sexes [43]. In the hemocytes (He) and cuticle (Cu), the expression of *Sp-CYP302a1* was significantly higher in female individuals compared to males, suggesting that this gene may have sex-specific functions in these tissues. The hemocytes plays an essential role in energy metabolism and oxygen transport, while the cuticle is crucial for the formation and renewal of the exoskeleton [44]. Moreover, the elevated expression in the cuticle may reflect a greater need for exoskeleton repair and regeneration during reproduction, which is critical for coping with the physical demands of the reproductive period. In other tissues, such as the Gill (Gi), hepatopancreas (Hep), muscle (Mu), gonads (OV/Te), antennal gland (TG), and Y organ (YO), there were no significant differences in the expression levels between males and females. This suggests that the function of the *Sp-CYP302a1* gene in these tissues may be fundamental and not influenced by sex. For instance, the hepatopancreas, being a primary digestive and metabolic organ, has broad essential functions that likely lead to similar metabolic demands in both males and females [45]. The lack of significant sex differences in the gonads suggests that *Sp-CYP302a1* plays a role in maintaining fundamental gonadal functions rather than participating in sex-specific physiological processes.

## 5. Conclusions

This study identified and analyzed the genes involved in the metabolism of 20-hydroxyecdysone (20E) in *S. paramamosain*, revealing the roles of different genes in 20E biosynthesis and metabolism. *CYP306a1* and *CYP314a1* may be functionally compensated by other genes from the P450 family. The significant expression of *Sp-Nvd*, *Sp-CYP302a1*, and *Sp-CYP315a1* in the Y-organ supports their critical roles in ecdysteroid biosynthesis, while the function of *Sp-CYP307a1* and *Sp-CYP18a1* are still ambiguous to some extent, which deserves further explorations. This study provides new insights into the diversity and regulatory mechanisms of ecdysteroids in crustaceans.

## Figures and Tables

**Table 1:** Oligonucleotide primers used in this study.

Name	Sequences (5′-3′)	Application
*Sp-Nvd-F*	GAAGTGAGTCTCGCGTTCCT	PCR
*Sp-Nvd-R*	GATGTGAAGTCAGCGGGACA	PCR
*Sp-CYP302a1-F*	GGCAGGTGTCTGAAGGAACA	PCR
*Sp-CYP302a1-R*	ACATATTCACCTCAGCTAGTGC	PCR
*Sp-CYP315a1-F*	GAGTGGAGGAAGTACCGCAC	PCR
*Sp-CYP315a1-R*	AGGCTACAGACAGTTAAGAGGC	PCR
*Sp-CYP307a1-F*	ACTATCGTGCTGATGATGAT	PCR
*Sp-CYP307a1-R*	AAGCGAGAGTAAGAAGACAA	PCR
*Sp-CYP18a1-F*	TCAAGCAGGATGGCGGTAAT	PCR
*Sp-CYP18a1-R*	CGTGAATTGTTGGGTCTTTGC	PCR
*Sp-Nvd-RTF*	TGGAGCTTGCTATTCTACCTGG	qRT-PCR
*Sp-Nvd-RTR*	ACATCCGTCAGGTCCTTAACA	qRT-PCR
*Sp-CYP302a1-RTF*	TCTCACAGAGAGTGCCGAGA	qRT-PCR
*Sp-CYP302a1-RTR*	ATGCAGGCAACCAATCAACG	qRT-PCR
*Sp-CYP315a1-RTF*	CCTCTGCTTGCTCATAGACCT	qRT-PCR
*Sp-CYP315a1-RTR*	ACTTCCACAATCTCTTGGCGA	qRT-PCR
*Sp-CYP307a1-RTF*	CTCCTCTGTGGGAGGTTACG	qRT-PCR
*Sp-CYP307a1-RTR*	ACTCGGGCTTCTTGATGCAG	qRT-PCR
*Sp-CYP18a1-RTF*	CCTGCTCATGTCCGTGAGATT	qRT-PCR
*Sp-CYP18a1-RTR*	CTGGTTCACGGACGGTATGT	qRT-PCR

**Table 2:** Presence of genes associated with 20E metabolism in some arthropods. The parts enclosed in red represent the genes missing in this species.

Order	Diptera	Cladocera	Decapoda			
Family	Drosophilidae	Daphniidae	Varunidae	Portunidae		Penaeidae
Species	*Drosophila melanogaster*	*Daphnia pulex*	*Eriocheir sinensis*	*Portunus trituberculatus*	*Scylla paramamosain*	*Litopenaeus vannamei*
*Neverland*	+	+	+	+	+	+
*CYP307a1*	+	+	+	+	+	+
*CYP306a1*	+	+	+	−	−	+
*CYP302a1*	+	+	+	+	+	+
*CYP315a1*	+	+	+	+	+	+
*CYP314a1*	+	+	−	−	−	−
*CYP18a1*	+	+	+	+	+	+

**Table 3:** Basic information about genes associated with 20E metabolism.

Gene Name	NCBI GenBank Accession ID	Number of Amino Acids/aa	Molecular Mass/Da	Theoretical pI	Similarity to Drosophila Genes	Subcellular Localization	Function
*Neverland*	MN782365	463	52,366.98	8.39	38.65%	Nucleus	Catalyzing the conversion of cholesterol to 7-dehydrocholesterol
*CYP307a1*	NC_087153	520	59,495.28	5.8	49.44%	ER	Involved in ecdysteroid synthesis
*CYP315a1*	MN782364	410	45,763.7	6.74	49.13%	ER	Catalytic hydroxylation of cholesterol ring C2
*CYP302a1*	NC_087179.1	538	61,138.29	8.76	36.25%	ER	Synthesize ecdysteroids to regulate genes related to growth and molting
*CYP18a1*	MN542780	655	74,730.67	8.4	39.60%	ER	Catalytic C-26 hydroxylation of 20E

**Table 4:** Secondary structure prediction of 20E metabolism proteins.

Protein	Percentage/%			
	α-Helix	Extended Strand	β-Turn	Random Coil
*Neverland*	34.56	15.77	5.62	44.06
*CYP307a1*	30.44	8.02	8.97	52.57
*CYP315a1*	48.78	7.56	2.44	41.22
*CYP302a1*	45.85	12.78	3.65	37.72
*CYP18a1*	47.81	8.95	5.14	38.10

## Data Availability

The original contributions presented in the study are included in the article/Supplementary Materials, further inquiries can be directed to the corresponding author.

## Supplementary Materials

The following supporting information can be downloaded at: https://www.mdpi.com/article/10.3390/genes15121586/s1, Table S1: Accession ID for amino acid sequences in sequence alignment.
