# Supplementary material for: Identification and Evolution Analysis of the Genes Involved in the 20-Hydroxyecdysone Metabolism in the Mud Crab, Scylla paramamosain: A Preliminary Study
Source: Genes (Basel). 2024 Dec 10;15(12):1586. doi: 10.3390/genes15121586 (PMC11675983; doi:10.3390/genes15121586)
Supplement: Supplementary file 1 [file genes-15-01586-s001.zip › genes-3298972-supplementary.pdf]

Table S1. Accession ID for amino acid sequences in sequence alignment

Gene Name	Species Name	Accession ID
Neverland	*Scylla paramamosain*	QNQ17531.1
	Drosophila melanogaster	NP_001097670.1
	Homarus americanus	KAG7164135.1
	Cherax quadricarinatus	XP_053646030.1
	Penaeus vannamei	XP_027231585.1
	Penaeus monodon	XP_037784246.1
	Hyalella azteca	XP_018017327.1
	Dicentrarchus labrax	XP_051269067.1
	Epinephelus fuscoguttatus	XP_049460320.1
	Crassostrea virginica	XP_022327487.1
CYP315a1	*Scylla paramamosain*	XM_064004062.1
	Saqmariasus verreauxi	QBJ27553.1
	Homarus americanus	XP_042236925.1
	Cherax quadricarinatus	XP_053628938.1
	Penaeus vannamei	XP_027238669.
	Portunus trituberculatus	XP_045131453.1
	Eriocheir sinensis	XP_050727638.1
CYP307a1	*Scylla paramamosain*	XII91307.1
	Portunus trituberculatus	XP_045129380.1
	Eriocheir sinensis	XP_050690573.1
	Sagmariasus verreauxi	QBJ27549.1
	Procambarus clarkii	XP_045597726.1
	Propylea japonica	UYU26124.1
	Cordylochernes scorpioides	UYV80312.1
CYP302a1	*Scylla paramamosain*	QNQ17529.1
	Portunus trituberculatus	XP_045105768.1
	Eriocheir sinensis	XP_050729196.1
	Sagmariasus verreauxi	QBJ27551.1
	Homarus americanus	XP_042223672.1
	Helicoverpa armigera	WRX06077.1
	Bombyx mori	XP_020718318.1
	Orussus abietinus	XP_012282602.1
	Cordylochernes scorpioides	UYV81845.1
CYP18a1	*Scylla paramamosain*	QPZ89042.1
	Procambarus clarkii	XP_045588376.1
	Cherax quadricarinatus	XP_053627893.1
	Homarus americanus	XP_042232456.1
	Sagmariasus verreauxi	QBJ27550.1
	Penaeus monodon	XP_037783897.1
	Penaeus chinensis	XP_047470769.1
	Penaeus indicus	XP_063586468.1

	Penaeus vannamei	XP_027215960.1
	Neocaridina denticulata	AIY69132.1
	Eriocheir sinensis	XP_050692357.1
